# The negative psychological impact of COVID-19 pandemic on mothers of children with attention deficit hyperactivity disorder (ADHD): a cross-section study

**DOI:** 10.1186/s43045-021-00139-z

**Published:** 2021-09-03

**Authors:** Amira Mohamed Yousef, Mohammad Gamal Sehlo, Amany Elshabrawy Mohamed

**Affiliations:** Department of Psychiatry, Faculty of Medicine, Zagazig University, Zagazig, Egypt

**Keywords:** COVID-19, ADHD, ADHD mothers, Stress, Anxiety, Depression

## Abstract

**Background:**

ADHD is an important cause for psychiatric care as one of the most prominent neurodevelopmental conditions. Being an ADHD child’s parent is a daunting and sometimes stressful job that becomes more challenging during the COVID-19 pandemic and its negative consequences. This cross-sectional study was applied to 54 mothers of ADHD children and adolescents; the data of the ADHD children and adolescents have been registered before the lockdown on their regular follow-up visits on the child and adolescent’s clinic of the psychiatry department. Data of the study were collected through an electronic Google form included a COVID-19 questionnaire, The Depression, Anxiety, and Stress Scale—21 Items (DASS-21) (Arabic version), and Conners’ Parent Rating Scale Revised-short version (CPRS-48). We aimed to assess symptoms of anxiety, depression, and stress symptoms among the mothers of ADHD children and adolescents during COVID-19 outbreaks and determine the relationship between these symptoms and the changed circumstances that occurred by the COVID-19 pandemic, also with the behavioral problem of their children that may deteriorate by their mothers’ psychological state.

**Results:**

Most of our participants were from the low socioeconomic class and were married. In total, 29.6% of the mother of ADHD children had confirmed COVID-19 cases among their family members, while 11.1% of our subjects lost a family member due to this epidemic, 22% of our sample lost their work because of COVID-19. We found that the COVID-19 pandemic has a big financial drawback on the family of our participants by a percentage of 31.5%. We noticed that 48.1% of our sample documented domestic violence toward them compared to before COVID-19. A total of 92.6% of the mothers who participated in our study assessed the period of change and restrictions as being very demanding. We found that 74.1% of ADHD patients were not compliant with their medications than before the epidemic. Also, we found that the mothers of ADHD children have bigger challenges in managing the child’s meals, structured activities, and sleep compared to before COVID-19. We showed that 53.7% of the mothers had depressive symptoms, 61% had anxiety symptoms, and 53.7% had stress symptoms. These symptoms were statistically associated with the lost family member due to COVID-19, the financial drawbacks of the COVID-19 outbreak, and the domestic violence toward mothers, compared to before COVID-19, the non-compliance of their children on the medications compared to before COVID-19, the presence of confirmed COVID-19 cases among family members, mother’s assessment of the period of change and restrictions as being very demanding compared to before COVID-19, and the non-compliance of their children on the medications compared to before COVID-19 as well as the increased behavioral symptoms of their children.

**Conclusion:**

COVID-19 pandemic has psychological influences on mothers of children with ADHD. A significant number of them may have depression, anxiety, and stress symptoms that could affect their children’s compliance with the medication and, consequently, their symptomatology.

## Background

The 2019 coronavirus pandemic (COVID-19) affects life deeply all over the world. The lockdown constraints and associated economic closings create a dramatic alteration in the population’s social environment. These policies could harm the children and adolescents’ mental health markedly. An increased risk for a parental psychiatric condition, domestic violence, and childhood abuse can also pose a significant threat. Particularly, for disabled children and adolescents [1].

ADHD is an important cause for referral to psychiatric care as one of the most prominent neurodevelopmental conditions. Though inattention and hyperactivity/impulsivity are the major symptoms of the disorder [2]. It involves a wide variety of symptoms and impairment in functioning [3]. Besides that, these impairments may be either mitigated or aggravated by environmental influences [4]. ADHD can impact children’s social, cognitive, and academic abilities. These children need adequate therapies to avoid facing difficulties both at home and in the community in general [5]. These psychosocial disabilities can impact the quality of life of both individuals and family, which go beyond the main effects of attention deficit, hyperactivity, and impulsivity [6]. Being an ADHD child’s parent is a daunting and sometimes stressful job. Dealing with these children’s developmental issues will intensify parental inadequacies and social challenges. Managing multiple ADHD issues can have a negative impact on the mental health of parents [7].

Mothers undergo life events that they consider unmanageable and judge parenting stress as severe. Gerdes et al., in their study, assumed that experiencing these events and, as a result, having increased pressure led mothers to be unable to react to the child’s negative behavior [8]. This interferes with the ability of the mother to solve problems and further enhancing their depressive symptoms. Related to these theories, being in the COVID-19 pandemic is a major life-threatening problem. And as the coronavirus pandemic progresses quickly worldwide, there is a significant amount of uncertainty, worry, and concern in the general populace and, precisely, among some populations, such as older adults, caregivers, and people with pre-existing medical or mental conditions. To date, excessive levels of depression and anxiety are the significant psychiatric consequences of public mental health. However, recent interventions, especially quarantine and its effects on many people’s habits, schedules, or livelihoods——are also predicted to increase anxiety, stress, depression, unhealthy alcohol and substance use, and self-harm or suicidal behavior [9]. Quarantine brings an overload of psychological stress, several neuropsychiatric symptoms [10]. Home isolation has an acute and lasting psychosocial effect on children, particularly ADHD children, because of the significant changes in their lifestyle and physical activity [11].

Parenting is a lengthy, complicated process that constrains a parent’s ability to live a certain lifestyle even before the child is born. Raising an infant with ADHD creates extra difficulties and challenges due to the disturbed behavior of the child [7]. Numerous studies have found that parents with children with ADHD face greater stress than parents with children without ADHD [12–14].

Furthermore, parents with children with ADHD showed more parental stress than parents with children with other serious conditions like epilepsy and autism [15, 16]. This association is not only understood by the manifestations of ADHD themselves but also explained by the multiple issues associated with child ADHD, including oppositional defiant disorder or learning disabilities [17, 18]. Since the COVID-19 pandemic and its negative consequences, this has become more complicated. Children with ADHD were not likely to get adequate educational help. Many of these children’s parents may be considered not to be experts in the field of education. Still, they are forced to be responsible for educating their children in addition to struggling with all the children’s emotional and behavioral problems 24/7. Furthermore, the COVID-19 pandemic has been a big battle for everyone, including parents. Their anxiety about this event could even increase the psychological and behavioral problems of the children [19]. That is why incidents of child abuse, negligence, oppression, and domestic violence are on a horrid increase during the COVID-19 pandemic and its quarantine [20]. In this research, we intended to assess symptoms of anxiety, depression, and stress symptoms among the mothers of ADHD children and adolescents during COVID-19 outbreaks and determine the association between these symptoms and the changed circumstances that occurred by the COVID-19 pandemic, also with the behavioral problem of their children that may increase during the pandemic.

## Method

### Study site, design, and participant

This cross-sectional study was applied to the mothers of ADHD children and adolescents; the data of the ADHD children and adolescents have been registered before the lockdown during their regular follow-up visits to the Child and Adolescents Clinic of the Psychiatry Department of the Faculty of Medicine, Zagazig University Hospitals, Sharkia, Egypt.

The study was performed from May 21 to July 30, 2020.

The sample size was 54 mothers that were calculated by the OPEN EPI software package [21]. Assuming that percentages of mothers of ADHD patients having anxiety problems versus the control group were 43.7% versus 18%, at confidence level 95% and power 80%.

The mothers of ADHD patients’ group were eligible for this study according to specific criteria that included that they have children who met the criteria of ADHD according to DSM-5 aged from 6 to 17 years old without chronic medical illness or comorbid psychiatric disorders. Also, they do not have another child with a chronic medical condition or psychiatric disorder. We excluded all mothers with known chronic health problems or have a family history or a past history of psychiatric disorders. And mothers who cannot read or write the Arabic language or did not have sufficient electronic knowledge.

An electronic informed consent was obtained from all the participants before enrolling in this study by answering an obligatory Yes or No inquiry representing their acceptance or refusal to participate in our research.

### Study tools

First, we phoned the mothers who were eligible for the study to explain our study’s aim and procedure. Those who intended to participate were subsequently sent a text message, providing information about the study. Accordingly, this was a closed survey, which was sent to a particular group of mothers directly contacted by the research team. The participants were presented with the complete questionnaire using an electronic Google form which was immediately collected on an Excel sheet to be analyzed once the participants finished and submitted it. Only the study researchers could access the participants’ personal data that are hidden as data were analyzed.

The electronic Google form consisted of four sections:

1. The first section contained an explanation of the aim of our study, its procedures, and an obligatory Yes or No inquiry representing the participants’ acceptance or refusal to participate in our research.
2. The second section involved a COVID-19 questionnaire included questions by which we collected the sociodemographic data (age and sex of the child of the participant mothers, age of the participant mothers, residency, social class, marital status, and the number of siblings), also included questions to determine changing circumstances related to COVID-19 epidemic (presence of COVID-19 among family members, bereavement due to COVID-19). Parent’s present working state, presence of violence toward the mother as compared to before COVID-19, the financial impact of COVID-19 outbreak on the family, the mothers’ judgment on this period of change and restrictions, the compliance of the ADHD children on treatment as compared to before COVID-19, and if there were more significant challenges in the management of the child’s structured activities, diets, and sleeping as compared to before COVID-19.

The financial impact of the COVID-19 outbreak on the family was measured by the decrease in the family income and the degree of its impact on their daily living.

The mothers were judging this period of change and restrictions according to the burden she had to bear by taking care of her children 24/7 including their studying and the free time she could have for her own and her accommodation for being unable to go out as usual.

We meant by the child’s structured activities, the activity that is planned and intentionally directed by an adult like studying, drawing, reading, and structured playing.

3- The third section involved The Depression, Anxiety and Stress Scale—21 Items (DASS-21) (Arabic version) [22, 23]. It is a quantitative measurement scale of depression, anxiety, and stress symptoms (each calculated by seven phrases) over the previous week. The subscales measure depression symptoms (e.g., life was meaningless), anxiety symptoms (e.g., trouble relaxing), and overall stress symptoms (e.g., feeling rather touchy). Every mother involved in the research should decide how intensely each of the statements of scales applies to her with a score from zero to three where 0 is “never felt,” 1 is “feels it to a degree or some degree,” 2 is “feels it to a substantial amount or to a great deal of time,” and 3 is “feels it too much and most of the time.” The scaling scores are as follows: (0-9) for depression is recognized to be normal, mild (10-13), moderate (14-20), and severe and very severe cases are scored (≥ 21), (0-7 for anxiety) are accepted as normal; (8-9) for mild cases, moderate to very severe cases are scored (≥ 15), and scoring stresses; (0−14) are considered normal, and (15-18) for mild cases, (19−25) scores means moderate cases while severe to excessively severe cases (≥ 26). The Arabic translation was handled by Taouk Moussa et al. [24]. An Egyptian study announces the findings of a differential item functioning analysis of the DASS-21 in a group of Egyptian drug consumers with a coefficient alpha of 0.883 and inter-item correlations of (0.249) [25]. In our study, the (DASS-21) scale (Arabic version) was tested for content validity by two panels of the Psychiatric Department experts. These experts assessed the tool for clarity, relevance, comprehensiveness, applicability, and understanding.
  The reliability of the scale was tested by measuring its internal consistency. It demonstrated an excellent level of reliability (Cronbach’s alpha = 0.84).
4- The fourth section included Conners’ Parent Rating Scale Revised-short version (CPRS-48) (to assess the behavioral problem of their children during the COVID-19 pandemic). This short assessment scale consisted of six subscales to evaluate (a) conduct problems (eight items), (b) learning problems (four items), (c) psychosomatic problems (four items), (d) impulsive hyperactivity (four items), and (e) anxiety (four items), and (f) the hyperactivity index (10 items). Parent(s) rated their child’s behavior in the previous month on a four-point scale ranging from 0 (not at all) to 3 stars (severely affected). Questionnaires usually take 30–45 min to finish. Each question’s score is converted to a *t* score with an average of 50 and a standard deviation of 10. The child has a behavioral problem whether his or her *t* score is two SDs higher than the average [26]. El-Sheikh et al. translated the **A**rabic version of the scale [27]. Test-retest and inter-rater reliability were 0.64 and 0.68, respectively [28].

### Statistical analysis

SPSS program version 27 was used for statistical analysis (IBM, 2020). Tables were used to display the information. The mean, median, standard deviation, and range were used to present quantitative results. Frequencies and proportions were used to present qualitative results. The Shapiro-Wilk test was used to assess the variance homogeneity and distribution properties of variables. To evaluate qualitative variables, Pearson’s chi-squared test, Fisher’s exact test, and chi-square for linear trend were used as required.

The Mann Whitney *U* (MW) test and the Student’s *t* test were used to comparing quantitative variables between two classes, as indicated.

A *P* value of ˂ 0.05 was considered statistically significant [29].

## Results

### Results of sociodemographic data

Most of our sample were married and from the low socioeconomic class and live in the urban area. The mean age of the mothers was 30.8 ± 4.56 (Table 1).

**Table 1:** Demographic and other characteristics of the study participants

Variables	Study participants (***n*** = 54)
Age of child of the participant mothers (years)	
Mean ± SD	10.3 ± 3.1
Age of the participant mothers (years)	
Mean ± SD	30.8 ± 4.56
Median	22-40.6
Sex of the child of the participant mothers, *n* (%)	
Male	38 (70.4%)
Female	16 (29.6%)
Residence, *n* (%)	
Urban	48 (88.9%)
Rural	6 (11.1%)
Social class, *n* (%)	
Low	24 (44.4%)
Middle	18 (33.3%)
High	12 (22.2%)
Marital status, *n* (%)	
Married	46 (85.2%)
Divorced	5 (9.3%)
Widow	3 (5.5%)
The number of siblings, *n* (%)	
Median (range)	2 (1-4)
Presence of confirmed COVID-19 cases among family members, *n* (%)	
Yes	16 (29.6%)
No	38 (70.4%)
Lost family member due to COVID-19, *n* (%)	
Yes	6 (11.1%)
No	48 (88.9%)
Father’s present working state, *n* (%)	
Regularly commuting to work	32 (59.3%)
Smart working	13 (24.1%)
Not working because of COVID-19	8 (14.8%)
Not working since before COVID-19	1 (1.9%)
Mother’s present working state, *n* (%)	
Not working because of COVID-19	22 (40.7%)
Not working since before COVID-19	14 (25.9%)
Working from home	11 (20.4%)
Regularly commuting to work	7 (13.0%)
Financial drawbacks of COVID-19 outbreak, *n* (%)	
No drawbacks	16 (29.6%)
Little drawbacks	21 (38.9%)
Big drawbacks	17 (31.5%)
Domestic violence toward the mother as compared to before COVID-19, *n* (%)	
Present	26 (48.1%)
Absent	28 (51.9%)
Mother’s assessment of the period of change and restrictions as compared to before COVID-19, *n* (%)	
Very demanding	50 (92.6%)
Demanding	4 (7.4%)
Not demanding	0 (0.0%)
The compliance of the ADHD children on treatment as compared to before COVID-19	
Compliant	14 (25.9%)
Non-compliant	40 (74.1%)
Bigger challenges in the management of the child’s meals as compared to before COVID-19	31 (57.4%)
Bigger challenges in the management of the child’s structured activities as compared to before COVID-19	40 (74.1%)
Bigger challenges in the management of the child’s sleep as compared to before COVID-19	48 (88.9%)

### Results related to the changing environment caused by the COVID-19 epidemic

- We found that 29.6% of our sample had confirmed COVID-19 cases among their family members, while 11.1% lost a family member due to this epidemic (Table 1).
- We found that 22% of the participating mothers lost their work because of COVID-19, while 14.8% of the fathers lost their job because of COVID-19 (Table 1).
- We found that the COVID-19 pandemic has a big financial drawback on 31.5% of our participants (Table 1).
- We noticed that 48.1% of our sample documented the presence of domestic violence toward them compared to before COVID-19 (Table 1).
- A total of 92.6% of the mothers who participated in our study assessed the period of change and restrictions compared to before COVID-19 as being very demanding (Table 1).
- In total, 74.1% of the ADHD children of the participating mothers were not compliant with their medications as compared to before COVID-19.
- Our results showed that 57.4%, 74.1%, and 88.9% of the mothers of ADHD children had more significant challenges in managing the child’s meals, structured activities, and sleep compared to before COVID-19, respectively (Table 1).

### Results of The Depression, Anxiety and Stress Scale—21 Items (DASS-21) of the studied mothers

- We showed that 53.7% of the participating mothers had abnormal depression scores in the DASS-2, indicating the presence of depressive symptoms (Table 2).
- We showed that 61% of the participating mothers had abnormal anxiety scores in the DASS-2, indicating the presence of anxiety symptoms (Table 2).
- We found that 53.8% of the participating mothers had abnormal stress scores in the DASS-21, indicating the presence of stress symptoms (Table 2).

**Table 2:** The Depression, Anxiety and Stress Scale—21 Items (DASS-21) of the studied mothers

Items	Study participants (***n*** = 54)
Depression score	
Median (range)	8 (3-22)
• Normal	25 (46.3%)
• Mild	11 (20.4%)
• Moderate	16 (29.6%)
• Severe	2 (3.7%)
Anxiety score	
Median (range)	6 (2-16)
• Normal	21 (39.0%)
• Mild	20 (37.0%)
• Moderate	10 (18.5%)
• Severe	3 (5.5%)
Stress score	
Median (range)	12 (5-27)
• Normal	25 (46.3%)
• Mild	14 (25.9%)
• Moderate	12 (22.3%)
• Severe	3 (5.6%)

### Results of the association between depressive symptoms of mothers of ADHD children and other characteristics detected in the study

We showed that there was a statistically significant association between the mother’s depressive symptoms, and the male sex (*p* < 0.001*), the low social class (*p* < 0.001*), being divorced or widow) (*p* = 0.005*), the larger number of siblings) (*p* = 0.003*), presence of a lost family member due to COVID-19 (*p* = 0.03*), the father being not working as a result of COVID-19 (*p* < 0.001*), the presence of big financial drawbacks (*p* < 0.001*), and the domestic violence toward the mother compared to before COVID-19 (*p* < 0.001*), the mother’s assessment of the period of change and restrictions as being very demanding (*p* = 0.007*), the bigger challenges in managing the child’s meals than before COVID-19 (*p* = 0.032*), and the non-compliance of the ADHD children on treatment compared to before COVID-19 (*p* = 0.004*) (Table 3).

**Table 3:** Association between depression and other characteristics in mothers of ADHD children

Variables	Depression		***P***
	Yes (***n*** = 29)	No (***n*** = 25)	
Age of child (years)			
Mean ± SD	10.3 ± 3.3	9.8 ± 2.6	0.5
Sex of child, *n* (%)			
Male	26 (89.7%)	12 (48.0%)	< 0.001*
Female	3 (10.3%)	13 (52.0%)	
Residence, *n* (%)			
Urban	27 (93.1%)	21 (84.0%)	0.3
Rural	2 (6.9%)	4 (16.0%)	
Social class, *n* (%)			
Low	20 (69.0%)	4 (16.0%)	< 0.001*
Middle	6 (20.7%)	12 (48.0%)	
High	3 (10.3%)	9 (36.0%)	
Marital status, *n* (%)			
Married	21 (72.4%)	25 (100%)	0.005*
Divorced and widow	8 (27.6%)	0 (0.0%)	
The number of other siblings, *n* (%)			
Median (range)	3 (2-4)	2 (1-4)	0.003*
Presence of confirmed COVID-19 cases among family members, *n* (%)			
Yes	8 (27.6%)	8 (32.0%)	0.7
No	21 (72.4%)	17 (68.0%)	
Lost family member due to COVID-19, *n* (%)			
Yes	6 (20.7%)	0 (0.0%)	0.03*
No	23 (79.3%)	25 (100%)	
Father’s working status, *n* (%)			
Go to work as usual	3 (10.3%)	24 (96.0%)	< 0.001*
Work from home	12 (41.4%)	1 (4.0%)	
Not working since before COVID-19 or as a result of COVID-19	14 (48.3%)	0 (0.0%)	
Mother’s working status, *n* (%)			
Lost work as a result of COVID-19	10 (34.5%)	12 (48.0%)	0.3
Not working since before COVID-19	7 (24.1%)	7 (28.0%)	
Work from home	6 (20.7%)	5 (20.0%)	
Go to work as usual	6 (20.7%)	1 (4.0%)	
Financial drawbacks of COVID-19 outbreak, *n* (%)			
No drawbacks	2 (6.9%)	14 (56.0%)	< 0.001*
Little drawbacks	11 (37.9%)	10 (40.0%)	
Big drawbacks	16 (55.2%)	1 (4.0%)	
Domestic violence toward the mother as compared to before COVID-19, *n* (%)			
Present	25 (86.2%)	0 (0.0%)	< 0.001*
Absent	4 (13.8%)	25 (100%)	
Mother’s assessment of the period of change and restrictions as compared to before COVID-19, *n* (%)			
Very demanding	29 (100%)	15 (60.0%)	0.007*
Demanding	0 (0.0%)	9 (36.0%)	
Not demanding	0 (0.0%)	1 (4.0%)	
The compliance of the ADHD children on treatment as compared to before COVID-19, *n* (%)			
Compliant	3 (10.34%)	11 (44%)	0.004*
Non-compliant	26 (89.66%)	14 (56%)	
Bigger challenges in the management of the child’s meals as compared to before COVID-19			
Yes	20 (68.9%)	10 (40%)	0.032*
No	9 (31.1%)	15 (60%)	
Bigger challenges in the management of the child’s structured activities as compared to before COVID-19			
Yes	21 (72.4%)	12 (48%)	0.066
No	8 (27.6%)	13 (52%)	
Bigger challenges in the management of the child’s sleep as compared to before COVID-19			
Yes	19 (65.5%)	11 (44%)	0.112
No	10 (34.5%)	14 (56%)	

Tests of significance: Student’s *t* test, Pearson’s Chi-square test, Mann-Whitney *U* test

*Statistically significant (*p* value < 0.05)

### Results of the association between anxiety symptoms of mothers of ADHD children and other characteristics detected in the study

We identified a statistically significant association between the mother’s anxiety symptoms and the older age of the child (*p* = 0.04*), being divorced or widow (*p* = 0.01*), the larger number of siblings (*p* < 0.001*) (*p* = 0.001*), the working status of both fathers (*p* = 0.004*), and mothers (*p* < 0.001*), the mother’s assessment of the period of change and restrictions as being very demanding (*p* = 0.01*), the bigger challenges in the management of the child’s structured activities compared to before COVID-19 (*p* = 0.006*), and the non-compliance of the ADHD children on treatment compared to before COVID-19 (*p* = 0.017*) (Table 4).

**Table 4:** Association between anxiety and other characteristics in mothers of ADHD children

Variables	Anxiety		***P***
	Yes (***n*** = 33)	No (***n*** = 21)	
Age of child (years)			
Mean ± SD	11.0 ± 3.2	9.4 ± 2.0	0.04*
Sex of child, *n* (%)			
Male	25 (75.8%)	13 (61.9%)	0.3
Female	8 (24.2%)	8 (38.1%)	
Residence, *n* (%)			
Urban	30 (90.9%)	18 (85.7%)	0.6
Rural	3 (9.1%)	8 (14.3%)	
Social class, *n* (%)			
Low	15 (45.5%)	9 (42.8%)	0.6
Middle	12 (36.4%)	6 (28.6%)	
High	6 (18.1%)	6 (28.6%)	
Marital status, *n* (%)			
Married	25 (75.8%)	21 (100%)	0.01*
Divorced and widow	8 (24.2%)	0 (0.0%)	
The number of other siblings, *n* (%)			
Median (range)	3 (2-4)	1 (1-3)	< 0.001*
Presence of confirmed COVID-19 cases among family members, *n* (%)			
Yes	15 (45.5%)	1 (4.8%)	0.001*
No	18 (54.5%)	20 (95.2%)	
Lost family member due to COVID-19, *n* (%)			
Yes	5 (15.2%)	1 (4.8%)	0.2
No	28 (84.8%)	20 (95.2%)	
Father’s working status, *n* (%)			
Go to work as usual	15 (45.5%)	17 (81.0%)	0.004*
Work from home	13 (39.4%)	0 (0.0%)	
Not working since before COVID-19 or as a result of COVID-19	5 (15.1%)	4 (19.0%)	
Mother’s working status, *n* (%)			
Lost work as a result of COVID-19	5 (15.1%)	17 (81.0%)	< 0.001*
Not working since before COVID-19	10 (30.3%)	4 (19.0%)	
Work from home	11 (33.3%)	0 (0.0%)	
Go to work as usual	7 (21.2%)	0 (0.0%)	
Financial drawbacks of the COVID-19 outbreak, *n* (%)			
No drawbacks	10 (30.3%)	6 (28.6%)	0.2
Little drawbacks	10 (30.3%)	11 (52.4%)	
Big drawbacks	13 (39.4%)	4 (19.0%)	
Domestic violence toward the mother as compared to before COVID-19, *n* (%):			
Present	15 (45.5%)	10 (47.6%)	0.9
Absent	18 (54.5%)	11 (52.4%)	
Mother’s assessment of the period of change and restrictions as compared to before COVID-19, *n* (%) Very demanding Demanding Not demanding	31 (93.9%) 2 (6.1%) 0 (0.0%)	13 (61.9%) 7 (33.3%) 1 (4.8%)	0.01*
The compliance of the ADHD children on treatment as compared to before COVID-19, *n* (%)			
Compliant	7 (21.2%)	11 (52.4%)	0.017*
Non-compliant	26 (78.8%)	10 (47.6%)	
Bigger challenges in the management of the child’s meals as compared to before COVID-19			
Yes	20 (60.6%)	9 (42.8%)	0.202
No	13 (39.4%)	12 (57.2%)	
Bigger challenges in the management of the child’s structured activities as compared to before COVID-19			
Yes	22 (66.7%)	6 (28.5%)	0.006*
No	11 (33.3%)	15 (71.5%)	
Bigger challenges in the management of the child’s sleep as compared to before COVID-19			
Yes	25 (75.5%)	12 (57.2%)	0.151
No	8 (24.5%)	9 (42.8%)	

Tests of significance: Student’s *t* test, Pearson’s Chi-square test, Mann-Whitney *U* test

*Statistically significant (*p* value < 0.05)

### Results of the association between stress symptoms of mothers of ADHD children and other characteristics detected in the study

Our results identified a significant association between the mother’s stress symptoms and male sex (*p* = 0.006*), social class (*p* = 0.003*), being divorced or widow (*p* = 0.04*), the larger number of siblings (*p* = 0.003*), fathers working status (*p* < 0.001*), presence of big financial drawbacks (*p* < 0.001*), and domestic violence toward the mother compared to before COVID-19 (*p* < 0.001*), and mother’s assessment of the period of change and restrictions as being very demanding (*p* = 0.007*), the bigger challenges in the management of the child’s meals (*p* = 0.034*), structured activity (*p* = = 0.007*) compared to before COVID-19, and the non-compliance of the ADHD children on treatment compared to before COVID-19 (*p* = 0.032*) (Table 5).

**Table 5:** Association between stress and other characteristics in mothers of ADHD children

Variables	Stress		***P***
	Yes (***n*** = 29)	No (***n*** = 25)	
Age of child (years):			
Mean ± SD	10.6 ± 3.9	9.8 ± 3.1	0.4
Sex of child, *n* (%)			
Male	25 (86.2%)	13 (52.0%)	0.006*
Female	4 (13.8%)	12 (48.0%)	
Residence, *n* (%)			
Urban	26 (89.7%)	22 (88.0%)	0.8
Rural	3 (10.3%)	3 (12.0%)	
Social class, *n* (%)			
Low	19 (65.5%)	5 (20.0%)	0.003*
Middle	7 (24.1%)	11 (44.0%)	
High	3 (10.3%)	9 (36.0%)	
Marital status, *n* (%)			
Married	22 (75.9%)	24 (96.0%)	0.04*
Divorced and widow	7 (24.1%)	1 (4.0%)	
The number of other siblings, *n* (%)			
Median (range)	3 (2-4)	2 (1-4)	0.003*
Presence of confirmed COVID-19 cases among family members, *n* (%)			
Yes	7 (24.1%)	9 (36.0%)	0.3
No	22 (75.9%)	16 (64.0%)	
Lost family member due to COVID-19, *n* (%)			
Yes	5 (17.2%)	1 (4.0%)	0.1
No	24 (82.8%)	24 (96.0%)	
Father’s working status, *n* (%)			
Go to work as usual	3 (10.2%)	24 (96.0%)	< 0.001*
Work from home	13 (44.8%)	0 (0.0%)	
Not working since before COVID-19 or as a result of COVID-19	13 (44.8%)	1 (4.0%)	
Mother’s working status, *n* (%)			
Lost work as a result of COVID-19	9 (31.0%)	13 (52.0%)	0.09
Not working since before COVID-19	6 (20.7%)	8 (32.0%)	
Work from home	8 (27.6%)	3 (12.0%)	
Go to work as usual	6 (20.7%)	1 (4.0%)	
Financial drawbacks of the COVID-19 outbreak, *n* (%)			
No drawbacks	2 (6.9%)	14 (56.0%)	< 0.001*
Little drawbacks	10 (34.5%)	11 (44.0%)	
Big drawbacks	17 (58.6%)	0 (0.0%)	
Domestic violence toward the mother as compared to before COVID-19, *n* (%)			
Present	25 (86.2%)	0 (0.0%)	< 0.001*
Absent	4 (13.8%)	25 (100%)	
Mother’s assessment of the period of change and restrictions as compared to before COVID-19, *n* (%)			
Very demanding.	29 (100%)	15 (60.0%)	0.007*
Demanding	0 (0.0%)	9 (36.0%)	
Not demanding	0 (0.0%)	1 (4.0%)	
The compliance of the ADHD children on treatment as compared to before COVID-19, *n* (%)			
Compliant	9 (31.1%)	15 (60%)	0.032*
Non-compliant	20 (68.9%)	10 (40%)	
Bigger challenges in the management of the child’s meals as compared to before COVID-19			
Yes	22 (75.9%)	11 (44%)	0.034*
No	7 (24.1%)	14 (56%)	
Bigger challenges in the management of the child’s structured activities as compared to before COVID-19			
Yes	21 (72.4%)	9 (36%)	0.007*
No	8 (27.6%)	16 (64%)	
Bigger challenges in the management of the child’s sleep as compared to before COVID-19			
Yes	20 (68.9%)	12 (48%)	
No	9 (31.1%)	13 (52%)	0.117

Tests of significance: Student’s *t* test, Pearson’s Chi-square test, Mann-Whitney *U* test

*Statistically significant (*p* value < 0.05)

### Results of the association between the psychological state of the mothers of the ADHD cases and their children’s CPRS-48 scores

There was a significant association between the mother’s depressive symptoms and the higher scores in the conduct subscale (*p* = 0.007*), the psychosomatic subscale (*p* = 0.005*), the impulsive, hyperactive subscale (*p* = 0.006*), and the 10-items hyperactivity index subscales (*p* = 0.016*) of their children (Table 6).

**Table 6:** Association between the behavioral problem of ADHD children measured by Conners’ Parent Rating Scale Revised-short version (CPRS-48) and depression, anxiety, and stress symptoms of the mothers

Conners scale of ADHD children as compared to before COVID-19	Mothers’ depressive, anxiety, and stress symptoms measured by DASS-21								
	Depression			Anxiety			Stress		
	Yes (***n*** = 29)	No. (***n*** = 25)	***P***	Yes (***n*** = 33)	No. (***n*** = 21)	***P***	Yes (***n*** = 29)	No. (***n*** = 25)	***P***
Conduct score			0.007*			0.021*			0.06
Average	8 (27.6%)	16 (64%)		13 (39.4%)	15 (71.4%)		10 (34.5%)	15 (60%)	
High	21 (72.4%)	9 (36%)		20 (60.6%)	6 (28.6%)		19 (65.5%)	10 (40%)	
Learning score			0.06			0.029*			0.184
Average	10 (34.5%)	15 (60%)		12 (36.4%)	14 (66.7%)		11 (37.9%)	14 (56%)	
High	19 (65.5%)	10 (40%)		21 (63.6%)	7 (33.3%)		18 (62.1%)	11 (44%)	
Psychosomatic score			0.005*			< 0.001*			0.004*
Average	21 (72.4%)	25 (100%)		5 (15.2%)	17 (80.9%)		3 (10.34%)	11 (44%)	
High	8 (27.6%)	0 (0.0%)		28 (48.8%)	4 (19.1%)		26 (89.66%)	14 (56%)	
Impulsive hyperactive score			0.006*			0.202			0.007*
Average	25 (86.2%)	13 (52.0%)		13 (39.4%)	12 (57.1%)		8 (27.6%)	16 (64%)	
High	4 (13.8%)	12 (48.0%)		20 (60.6%)	9 (42.9%)		21 (72.4%)	9 (36%)	
Anxiety score			0.412			0.022*			0.202
Average	13 (44.8%)	14 (56%)		10 (30.3%)	13 (61.9%)		9 (31.1%)	12 (48%)	
High	116 (55.2%)	11 (44%)		23 (69.7%)	8 (38.1%)		20 (68.9%)	13 (52%)	
10-item hyperactivity index			0.016*			0.032*			0.016*
Average	8 (27.6%)	15 (60%)		9 (31.1%)	15 (60%)		7 (24.1%)	14 (56%)	
High	21 (72.4%)	10 (40%)		20 (68.9%)	10 (40%)		22 (75.9%)	11 (44%)	

Test of significance: Pearson’s Chi-square test

*Statistically significant (*p* value < 0.05)

Also, there was a significant association between the mothers’ anxiety symptoms and the high scores in the conduct subscale (*p* = 0.021*), the learning subscale (*p* = 0.029*), the psychosomatic subscale (*p* = <0.001*), the anxiety subscale (*p* = 0.022*), and the 10-items hyperactivity index subscales (*p* = 0.032*) of their children (Table 6).

While we found a significant association between the mother’s stress symptoms and the high scores in the psychosomatic subscale (*p* = < 0.004*), the impulsive hyperactive (*p* = 0.007*), and the 10-items hyperactivity index subscales (*p* = 0.016*) (Table 6).

### Results of the changes in Conners’ 3rd edition (PARENT short form) scale during COVID-19 pandemic compared to before the pandemic

**Fig. 1:**
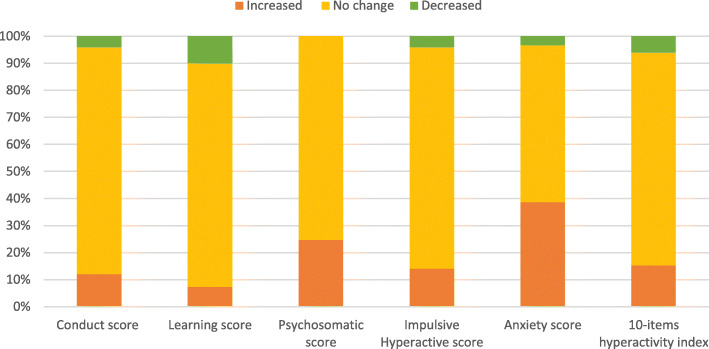
Change in Conners’ 3rd edition (PARENT Short Form) scale during COVID-19 pandemic compared to before the pandemic

Our results showed a significant increase in the conduct problem scores (*p* = 0.02*) as 12% of the children had increased conduct problem scores during the pandemic compared to before it. While 7.3% of the children showed an increase in the learning problem score, this was not significant (Fig. 1).

As regards the psychosomatic problem scores, our results showed a significant increase in the psychosomatic problem scores (*p* = < 0.001*), as 24.7% of the children had increased psychosomatic problem scores during the pandemic compared to before it (Fig. 1).

Moreover, our results showed a significant increase in the impulsive hyperactive problem score (*p* = 0.01*), anxiety problem score (*p* = < 0.001*), and the 10-items hyperactivity index score (*p* = 0.03*)] as 14%, 38.7%, and 15.35% of the children had increased in the impulsive hyperactive problem score, the anxiety problem score, and the 10-items hyperactivity index score, respectively, during the pandemic compared to before it (Fig. 1).

## Discussion

Attention deficit hyperactivity disorder is a pervasive and crippling disorder that affects multiple areas of life, including academic, social, and family relationships. Compared to the general population, children with ADHD are more likely to have parents with major interpersonal conflicts, family strife, and parental psychiatric conditions [30]. The complications linked to ADHD are further intensified by comorbid problems in more than 50% of the patients, including significant behavioral and emotional symptoms [31].

Coronavirus disease 2019 (COVID-19) pandemic resulted in a total shutdown in many countries, including Egypt. Subsequently, the schools were closed as a part of the lockout. Children are supposed to stay all the time inside their homes. It is understood that ADHD children can be particularly difficult to keep within the house in a condition like a lockout. They could be a continuous cause of disruption for family members, and families can also have difficulty managing them [32]. The study of Coghill et al. [33] and Yousefia et al. [34] believed that raising a child with ADHD is a great stress for parents. In normal circumstances, what about the frustrating situation associated with the COVID-19? We aimed in our study to assess symptoms of anxiety, depression, and stress symptoms among the mothers of ADHD children and adolescents during COVID-19 outbreaks and determine the relationship between these symptoms and the changed circumstances that occurred by the COVID-19 pandemic, also with the behavioral problem of their children that may deteriorate by their mothers’ psychological state. We conducted our study on 54 participants of the mothers of ADHD patients. Our research identified that (22%) of the participating mothers lost their work because of COVID-19, while 14.8% of the fathers lost their job because of COVID-19. Also, we found that the COVID-19 pandemic has a big financial drawback on the family of our participants by a percentage of 31.5%.

According to research carried by the Social Policy Institute at Washington University on 5500 participants in 2020, 24% lost their jobs or incomes as a result of COVID-19, and 46% of low-income individuals recorded trouble paying their mortgage after the epidemic started, with roughly 32% saying it was hard for them to support their families. Around 20% of middle-income individuals had to deal with these issues [35].

Our findings showed that 46.3% of our sample documented the presence of domestic violence toward them compared to before COVID-19. This was in line with the UN’s global alert about the rise in domestic violence toward females. In France, they recorded that abuse against women is said to have risen by 30% during the first episode of lockdown [36]. We showed a statistically significant association between the symptoms of depression and stress of our participating mothers and the domestic violence toward them, which was increased compared to before COVID-19. There is an epidemiological relationship between anxiety, depression, mental illness, adverse life experiences, and a bad relationship with a partner [37].

Our results showed that the mothers of ADHD children have bigger challenges in the management of the child’s meals, structured activities, and sleep as compared to before COVID-19, according to Owens JA, who recorded that sleep problems, especially in the beginning and sustaining sleep, are generally found in children and adolescents with ADHD [38]. Pagoto et al. [39] demonstrated that ADHD patients had difficulties monitoring their diet. Davis et al. reported that ADHD is positively correlated with “unhealthy” food consumption that is hard to handle [40].

We found that mothers of ADHD children have increased levels of depression, anxiety, and stress symptoms, which were associated with lost family members and the presence of significant financial drawbacks due to COVID-19, domestic violence toward the mother compared to before COVID-19, and the mother’s assessment of the period of change and restrictions as being very demanding.

Since the start of the COVID-19 pandemics, 45% of parents have reported increased caregiver burden, according to a study published in pediatrics. The percentage of parents who said they were in a bad mood part of the time increased from 10 to 33% of the time, and the number of parents who said they were in a bad mood all day increased from 9 to 29% of the time [41].

Moreover, the mother’s depressive, anxiety, and stress symptoms were associated with the non-compliance of the ADHD children on treatment compared to before the epidemic. Subsequently, we found an association between these symptoms and the increased conduct, learning, psychosomatic, impulsive hyperactive, anxiety problems in their children compared to before COVID-19, which was expected as many studies showed that internalizing and externalizing problems of the children increased in depressed mothers. Also, maternal depression affected the development of both conduct and depressive symptoms in ADHD children [37, 42].

A study showed that depression of the mothers unenabled them to respond correctly to the non-compliant behavior of their ADHD children result in more behavioral problems in those children with deterioration of their condition [43].

The study of Sanders identified that the mother’s psychological state affects her role and, subsequently, her children’s behavior. In one study, they found that anxious mothers were associated with hyperactivity and aggression among their children [44].

According to Yusefi’s research, some symptoms of ADHD were correlated with an increase in parent/child conflicts. Additionally, ADHD has been linked to maternal stress [45].

Costin discovered a strong correlation between hyperactivity in children and maternal anxiety, stress, and depression [46].

This study revealed that the period of change and restriction related to COVID-19 was associated with worsening of ADHD symptoms in the form of increased conduct, learning, impulsive hyperactive, and particularly the psychosomatic and anxiety problems in these patients during the pandemic as compared to before it. Furthermore, the problems remained in a large number of children. Just a few children showed any improvement in their behavioral difficulties over the lockdown time. ADHD patients have poor tolerance for ambiguity, making it difficult for them to follow instructions and comprehend the complexities of the pandemic scenario. According to Cortese et al., the imposed situation at home and the hostile setting, which alters their usual pattern, may raise the likelihood of more severe hyperactivity and impulsive behaviors. Also, this worsening of symptoms is consistent with the disorder’s nature and the constraints enforced as part of the shutdown [47].

In line of our results, Zhang and colleagues’ study found that children’s ADHD symptoms significantly deteriorated during the COVID-19 pandemic compared to their normal state [48]. Also, inconsistent with the study of Shah, R. and colleague, who found that the lockout caused by the COVID-19 pandemic was related to deteriorating the symptoms of ADHD in half of their study participants, which was in the form of a rise in the level of activity (slight or significant), irritability, or disruptive conduct behavior [49].

While the study of Bobo et al. found improvements in restlessness and duration of study time in connection with the relief of stress caused by the forced regularity of scholarly activities, although some parents identified worsening of the oppositional/defiant behavior and emotional outbursts. Parents also documented sleep disturbance and anxiety symptoms in their ADHD children. The authors attributed this improvement to the decreased school-related stress and schedules that accommodated their children’s rhythms. Improved self-esteem was another factor as their children were receiving less negative feedback [50].

In our opinion, the COVID-19 pandemic increased the presentation of depression, anxiety, and stress symptoms of the mothers of ADHD children by the changing that occurred in the surrounding circumstances like the financial impact, the increased domestic violence, and the burden of having confirmed COVID-19 cases or loses among family members. These psychiatric symptoms may affect the mother’s ability to care for their children, including the compliance of their children with their medication which was also affected by the financial drawbacks of the epidemic. These factors may deteriorate the ADHD symptoms of these children and adolescents that were already and directly affected by the stressful condition of the pandemic. So, as a psychiatrist, we should carefully observe the psychological state of the mothers of ADHD children, especially in a distressful situation like the pandemic, to give them appropriate psychiatric care and avoid the impact of their symptoms on their children’s condition.

### Limitation of the study

Although this study was the first one in Egypt to determine the psychological impact of the COVID-19 pandemic on a vulnerable group like the mothers of ADHD children, unfortunately, our research had some limitations, one of them is we did not have an assessment for depression, anxiety, and stress symptoms of the mothers of ADHD children before the COVID-19 epidemic to compare it with their evaluation after the pandemic, which in turn could determine the exact impact of it on our sample. Also, the cross-sectional design of this study failed to analyze the direction of the effect of the psychological state of the mothers on the behavioral symptoms of their children. Another limitation was the small sample size which is still less than expected according to previous studies. We were constrained by the available number of records in our clinic as the lockdown measures unenabled us to increase our sample size. Also, the lockdown measures prevented us from performing a structured psychiatric interview to diagnose the psychiatric disorders that may present in our participants or apply specific questionnaires instead of the subjective questions for assessment of certain points like the financial impacts and the domestic violence as this study was done through an electronic Google form questionnaire not face to face interview, so we were not able to use multiple questionnaires as many of our participants may find difficulty in completing it. So, we tried to make it as concise as possible and at the same time give us accurate data regarding our sample and what we try to assess in our study.

## Conclusion

COVID-19 pandemic has psychological influences on mothers of children with ADHD. A significant number of them may have depression, anxiety, and stress symptoms that could affect their children’s compliance with the medication and, consequently, their symptomatology.

## Data Availability

Upon request.

## Abbreviations

COVID-19: Coronavirus disease of 2019
ADHD: Attention-deficit hyperactivity disorder
DASS-21: The Depression, Anxiety, and Stress Scale—21 Items
CPRS-48: Conners’ Parent Rating Scale Revised-short version
MW: Mann-Whitney *U* test
*χ*^2^: Chi-square test
